# Study of Corrosion of Portland Cement Embedded Steel with Addition of Multi-Wall Carbon Nanotubes

**DOI:** 10.3390/ma18010210

**Published:** 2025-01-06

**Authors:** Miguel Angel Gómez-Aristizabal, Jhoan Mauricio Moreno-Vargas, Laura María Echeverry-Cardona, Elisabeth Restrepo-Parra

**Affiliations:** Laboratorio de Física del Plasma, Universidad Nacional de Colombia Sede Manizales, Campus la Nubia, Km 9 via al Magdalena, Manizales 170007, Colombia; mgomezar@unal.edu.co (M.A.G.-A.); jhmmorenova@unal.edu.co (J.M.M.-V.); lmecheverryc@unal.edu.co (L.M.E.-C.)

**Keywords:** structural cement, multi-walled carbon nanotubes, energy dispersion, corrosion, embedded steel

## Abstract

In this study, we research the innovative application of multi-walled carbon nanotubes (MWCNTs) as corrosion inhibitors in Portland cement embedded steel. The physicochemical properties of the dispersion solutions were evaluated, varying the storage time, to analyze their effect on corrosion resistance. Using a dispersion energy of 440 J/g and a constant molarity of 10 mM, stable dispersions were achieved for up to 3 weeks. These dispersions were characterized using Raman spectroscopy, UV-Vis spectroscopy and Zeta potential spectroscopy to assess the stability and structural damage of the MWCNTs. These results show that the addition of MWCNTs not only reduces the porosity of the cement matrix, but also forms an effective barrier against chloride ion intrusion, protecting the reinforcing steel. This approach stands out for combining improved mechanical properties and significant corrosion resistance, representing a promising innovation in the development of more durable construction materials.

## 1. Introduction

The study of composite materials is crucial in engineering due to their ability to combine different materials (polymers, metals and ceramics), resulting in unique properties. These materials are composed of a matrix (continuous phase), which defines the physical properties, such as surface finish and formability, as well as chemical properties, and a reinforcement (discontinuous or dispersed phase), which provides additional characteristics. A common example is concrete, where cement acts as a matrix and steel bars as reinforcement, increasing the durability of the structures [1].

However, concrete steel faces corrosion problems, with damages exceeding USD 100 billion [1]. The main causes are carbonation by carbon dioxide (CO_2_) and chloride attack, such as free chloride ions (Cl^−^) [2]. These factors affect the passive layer of the reinforcing steel, allowing electrochemical corrosion and compromising structural integrity [3].

Chloride ions (Cl^−^) trigger electrochemical corrosion by directly attacking the passive layer of steel, which is formed by thermodynamically more stable oxides such as Fe_3_O_4_ and Fe_3_O_3_ [2,4], generating iron oxide. This oxide causes the loss of optimal mechanical properties and a reduction in the resistance of steel and, hence, of the reinforced concrete utility [2]. The following is the stoichiometric relationship that describes the main oxidation process that steel experiences due to the intrusion of chloride ions:

Fe^2+^ + 2 Cl^−^→ FeCl_2_ → Soluble Salt (1)

FeCl_2_+ 2H_2_O → Fe(OH)_2_ + 2HCl (2)

where Fe(OH)_2_ is the oxide that increases the initial volume of the steel [2].

On the other hand, carbon dioxide (CO_2_) causes carbonation of concrete, which leads to a decrease in pH. When CO_2_ in the atmosphere reacts with moisture in the pores of concrete, it converts calcium hydroxide (Ca(OH)_2_), with a high pH, into calcium carbonate (CaCO_3_), with a more neutral pH. The stoichiometric ratio of carbonation is as follows [5]:

Ca(OH_2_) + CO_2_ **→** CaCO_3_ + H_2_O (3)

2CaO SiO_2_ 3H_2_O + 3CO_2_ **→** 2SiO_2_ + CaCO_3_ + 3H_2_O. (4)

3CaO Al_2_O_3_ 6H_2_O + 3CO_2_ **→** 2Al(OH_3_) + CaCO_3_ + 3H_2_O (5)

The above reactions occur in a relative humidity of 50–70% [5].

Concrete is generally highly alkaline (pH 12–13), which favors the protection of reinforcing steel from corrosion by forming a passive oxide layer on the steel surface. This layer remains stable in highly alkaline environments, but it is attacked by chlorides when they reach the exposed reinforced steel [5].

For these reasons, various studies have focused on preserving the passive layer on reinforcing steel, since the loss of this layer is the critical point between the initiation period and the propagation of corrosion [6,7,8]. The physical protection of concrete, which depends mainly on its porosity, is crucial to avoid or hinder the entry of aggressive agents, thus helping to prevent the spread of corrosion and wear of structures [1].

In response to this problem, various alternatives have been explored, such as the incorporation of doping agents and additives in cement to prevent the intrusion of chloride ions through its structure [9,10,11]. Among the materials used are nanosilica (NS) [9], silicon-based nanoparticles [1], graphite powder [12] and slag [10], obtaining promising results in terms of opposition to the penetration of corrosive agents, since it is shown that the reduced porosity of this material offered better stability conditions by preventing the penetration of aggressive agents [10].

Other studies have focused on the incorporation of multi-walled carbon nanotubes (MWCNTs), which have been shown to improve mechanical properties and reduce porosity in the cement matrix [13]. From an economic and practical perspective, the use of MWCNTs in Portland cement mixtures represents a strategic investment and an efficient solution to address critical problems in construction, such as corrosion degradation. By improving the durability of structures, as suggested by a few studies [13,14], the maintenance and repair frequency and costs are reduced for structures exposed to aggressive conditions, such as coastal or industrial areas. This results in long-term savings and an increased lifespan of constructions, promoting more sustainable practices. Furthermore, the incorporation of MWCNTs in Portland cement mixtures allows the reduction in cement porosity and reinforces the matrix, improving both the mechanical properties [13] and, possibly, the chemical resistance of the structures [14]. These advantages make them a viable and attractive alternative for high-performance applications, such as bridges, tunnels and buildings in harsh environments.

However, one of the greatest challenges in the incorporation of MWCNTs in cement specimens is their dispersion, since they tend to agglomerate instead of dispersing homogeneously [14]. To solve this problem, several techniques have been developed that include the use of ultrasound, which has proven to be effective for the homogeneous dispersion of MWCNTs in water before their addition to concrete [13]. Likewise, in other reports, the optimal parameters for the dispersion of MWCNTs were established, focusing on stability and homogeneity, achieving a stable dispersion with a concentration of 10 mM of TX-100 and an energy of 390 J/g [15].

Based on this work, the main objective of this study is to evaluate the electrochemical behavior of Portland cement embedded steel in samples with the addition of MWCNTs, using various characterization techniques: Zeta potential, Raman spectroscopy, UV-Vis spectroscopy, scanning electron microscopy and potentiostat–galvanostat. These characterization techniques are used to understand the criteria related to the electrostatic repulsion and attraction forces between particles [16] and to analyze the chemical and structural data [17], electronic and vibrational structures [18], the amount of radiation passing through the MWCNTs [19] and the morphology and chemical composition of the material with a resolution between 20 and 50 Å [20]. Finally, these specimens are tested using electrochemical tests in order to evaluate the corrosion in a material [21]. A constant current flow is applied through a load or resistance, irrespective of its specific value [22].

The progress in the research of construction materials has gained increasing relevance, especially for its significant impact on the daily life of society, because it focuses on improving the physical, chemical and mechanical properties of these materials as those that will be reviewed throughout this study. A particularly promising direction has been the exploration of carbon nanotubes, focusing on their ability to reinforce flexural strength and act as corrosion inhibitors, a crucial challenge for coastal structures, as suggested by studies [13,23].

In this context, this study is a novel contribution to the field of construction materials, showing how the integration of nanotechnology can optimize the performance and sustainability of modern infrastructures.

## 2. Methodology

### 2.1. Dispersion of MWCNTs

Aqueous dispersions containing 0.35% MWCNT (industrial grade NC7000 produced by Nanocyl) were prepared using TX-100 (M = 646.85 g/mol; density ρ = 1.060 ± 0.01 g/cm³) as a surfactant at a concentration of 10 mM. The surfactant was dissolved in deionized water (type 1: minimum resistivity at 20 °C of 19.5 MΩ and conductivity of 1 μS/cm) by magnetic stirring for 15 min at 680 rpm at room temperature. Subsequently, MWCNTs were added to this solution.

The mixture was subjected to sonication using a SONIC-250WT-110 sonicator from the Universidad Nacional de Colombia, Manizales Campus, applying 440 J/g of ultrasonic energy in 20-s on/off cycles to prevent overheating, using a 3 mm diameter tip. This methodology is based on the following study: Dispersion Energies and Surfactants in a Solution of Carbon Nanotubes in Water: Applications in Portland Cement Pastes.

**Figure 1:**
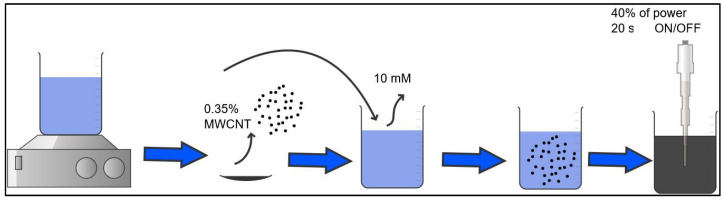
Preparation of dispersion.

Initially, 300 mL of a solution of water and 10 mM TX-100 were prepared; next, the MWCNTs (0.35% of the total mass) were added to the mixture. The total energy applied by the ultrasonic tip was 133,320 J, with a power of 100 J/s, corresponding to 40% of the maximum capacity of the equipment, according to the diameter of the tip available for this dispersion process (Figure 1).

It is worth noting that the specific energy delivered to the solution is higher than that reported in the literature. However, this is justified due to the smaller diameter of the ultrasonic tip used compared to the one commonly mentioned in the literature, which makes the energy delivery and effective dispersion of the MWCNTs more difficult. To compensate for this difference, the dispersion time is increased, which contributes to the increase in the total energy delivered. This decision is based on the following arguments:

A smaller ultrasonic tip diameter affects the dispersion efficiency of MWCNTs due to the smaller contact area and ability to generate cavitation energy.

Increasing the dispersion time and power input counteracts the limitation of the ultrasonic tip diameter.

An optimal dispersion process, achieved by adjusting sonication time and power, directly contributes to improving solution stability.

#### Dispersion Characterization

To carry out the measurements using ultraviolet–visible spectroscopy (UV-Vis), we have the support of the Laboratorio de Física del Plasma of the Universidad Nacional de Colombia, Sede Manizales, that has an MRC UV-61-PCS spectrometer (MRC Ltd., a company based in Holon, Israel). An aliquot of the solution is taken, which is diluted in water type 1 with TX-100, using a dilution factor of 1:100. In addition, a sample of water type 1 with TX-100 is prepared, which is magnetically stirred to be used as a reference target. The measurements are carried out in a range of 200 to 600 nm during weeks 1, 2 and 3 of storage, with the aim of observing the degree of dispersion.

To evaluate the stability of the data over time, Z potential measurements are carried out in diluted suspensions (0.01% by weight) at a temperature of 25 °C, with an absorption wave number of 0.019. The Zeta potential was measured at the DLS Hybrid Systems and Processes Intensification Lab of the Universidad Nacional de Colombia, Sede Manizales, using the LITESIZER 500 equipment (Anton Paa, a company based in Graz, Austria), which has a Zeta potential range of ±1000 mV. These measurements are carried out at weeks 0, 1, 2 and 3. At week 3, it is observed that the dispersion is in the unstable electric potential zone (+25 mV to −25 mV) for suspensions, and from this point forward, agglomerations in the dispersion are observable as the storage time increases. These lumps in the dispersion indicate particle agglutination [13], which means that there is no stability in the dispersion and that it is not homogeneous. It is necessary to have an efficient dispersion for its application and to guarantee a homogeneous distribution of MWCNTs in the cement. Therefore, the dispersions of week 0 and week 2 are used to prepare the specimens.

Raman spectroscopy is used to evaluate the damage to the MWCNTs caused by the energy applied during the sonication process. Raman spectra are measured using a LabRAM HR Evolution Horiba system (HORIBA France SAS, a company based in Palaiseau, France), from the associated research group Grupo de Tribología y Áreas of the Universidad Nacional de Colombia, Medellín campus, equipped with a 532 nm laser and a power of 3.20 mW. These measurements are carried out in week 2 of the first dispersion and in week 3 of the second dispersion.

### 2.2. Preparation of Test Tubes

The specimens are manufactured maintaining a ratio of 0.4 between the aqueous solution and the cement [12]. The aqueous solution refers to the dispersion of MWCNTs in water type 1 with TX-100, as well as the mixtures of only water type 1 with TX-100 and only water type 1. Before using the MWCNT dispersion to prepare the specimens, the solution is immersed in a bath in an ultrasonic cleaner (Labscient, a company based in Bogotá, Colombia), for 10 min.

**Figure 2:**
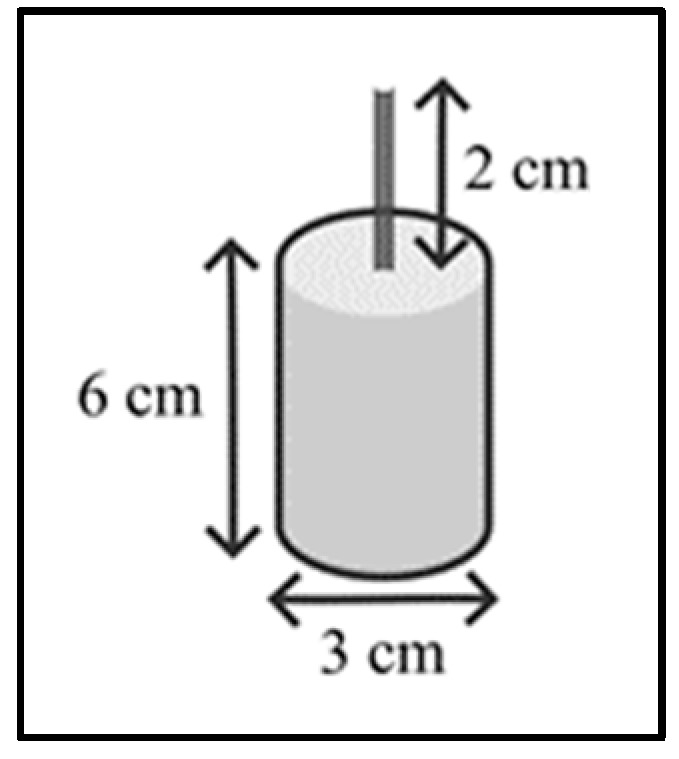
Specimen size.

For the preparation of the specimens, a cylindrical mold 3 cm in diameter and 6 cm in height, made of steel (Figure 2), is used, with the aim of maintaining a 2:1 ratio between the height and diameter of the specimens, as indicated in the NTC 1377 standard [24]. These dimensions do not represent a change in the dispersion of MWCNTs, since it is prepared separately, with the energy conditions mentioned in this report. The mixture was made according to the parameters of the NTC 1377 standard, which seeks to homogenize conditions in the mixture and to guarantee that the properties of the material are met. Likewise, it is expected that the engineering protocols for the preparation of cement mixtures in the construction industry are met to achieve a homogeneous dispersion of the MWCNT suspension in the structures [24].

Nine specimens were manufactured, according to a statistical design, to perform multiple measurements. The preparation of the specimens was carried out following the NTC 550 standard [25]. First, the cement is manually mixed with the solution in a container for 5 min, using a mixing rod, until a homogeneous mixture is obtained. Next, multipurpose oil is applied to the molds to prevent the cement from sticking to the walls. Then, the mold is filled, depositing the mixture in 3 equal layers using a compaction method that combines tamping and vibration [25].

**Figure 3:**
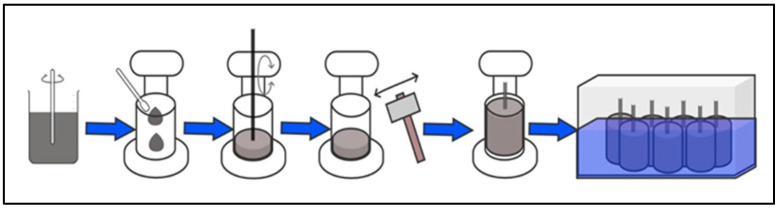
Preparation of test tubes.

The compaction process is carried out by depositing the cement mixture in the mold, which is tamped with a rod, applying 50 strokes per layer to ensure uniform distribution. To eliminate any air bubbles and cover holes, the edges of the mold are gently hit with a rubber hammer, repeating the process 50 times. Once the mold is full, an 8 cm corrugated bar (reinforcing steel) is inserted into the center of the specimen, so that it protrudes from the mold. Using a spatula, the excess cement is removed to obtain a homogeneous and smooth surface [25]. The samples are then removed from the mold after 24 h, and they undergo a curing process for 7, 14 and 28 days (Figure 3).

#### 2.2.1. Characterization of the Test Specimens

To evaluate the morphology of the different cement specimens, scanning electron microscopy (SEM) (TESCAN, a company based in Brno, República Checa), is used, with the support of the Rheology group (GReo) of the Department of Mechanical Engineering of the Pontifical Catholic University of Rio de Janeiro in Brazil. Images are obtained in a magnification range of (50–5) μm, where the cement matrix is observed in different states, depending on the additive present in each specimen.

#### 2.2.2. Induction of Deterioration

After each curing cycle, the specimens are immersed in a 3.5% NaCl solution for 7 days to expose them to an aggressive corrosion environment. While both sodium chloride (NaCl) and magnesium chloride (MgCl_2_) solutions are highly corrosive, NaCl is selected in this study due to its significantly higher abundance in marine environments compared to MgCl_2_, primarily due to the low concentration of the magnesium ion (Mg^2^⁺) in seawater. Moreover, Mg^2^⁺ ions can react with cement pastes, reducing pH and potentially increasing corrosion risk. According to the literature [10], the NaCl solution returns to non-aggressive values after 20 days, effectively simulating a critical corrosive maritime environment.

#### 2.2.3. Evaluation

The evaluation process of the specimens is carried out after 7 days of deterioration induction, through an analysis of the electrochemical behavior of the steel by measuring the resistance to linear polarization with a potentiostat–galvanostat, brand MetroOhm – DropSens (Metrohm DropSensOviedo, a company based in Asturias, España), model µStat 400, connected to a computer of the National University of Colombia, Manizales Campus.

**Figure 4:**
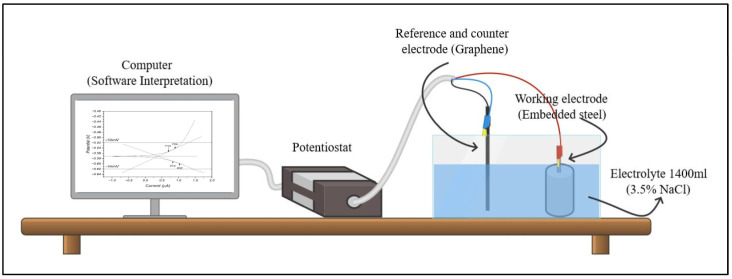
Mounting the potentiostat.

A three-electrode system is used, as shown in Figure 4. The reference electrode (blue head) and the counter electrode (black cable) are connected to a graphene bar that is used as a reference electrode, while the working electrode (red cable) is connected to the embedded steel, which is the research subject. An aqueous solution of 1400 mL volume with 3.5% NaCl is used as the electrolyte. To ensure data reproducibility, a total of nine independent measurements were performed for each sample type, using a suitable statistical design that allows the analysis of the variance in the data. This approach allowed to rigorously assess the variability of the results and to confirm the reliability of the data obtained.

The polarization resistance curve (Rp) is obtained by polarizing the working electrode at ±0.25 V with respect to the corrosion potential (Ecorr) at a standard sweep rate of 0.001 V/s. The corrosion analysis of the obtained curve is performed using the Drop View 8400 software by defining the parameters of density (of the steel) *ρ* = 7.85 g/cm^3^, molecular weight (according to the composition of the rebar) = 55.658 g/mol and immersed area (of the rebars in the electrolyte) = 11.79 cm^2^. Using this technique, it is possible to determine the corrosion current, the current density and, subsequently, the corrosion rate, which helps to understand how concrete affects the electrochemical behavior of the embedded steel.

It is important to note that this process is repeated for the second dispersion; the analysis aims to evaluate how the storage time of the dispersion influences the propagation of corrosion in Portland cement embedded steel.

## 3. Results and Discussion

### 3.1. Raman Spectroscopy

**Figure 5:**
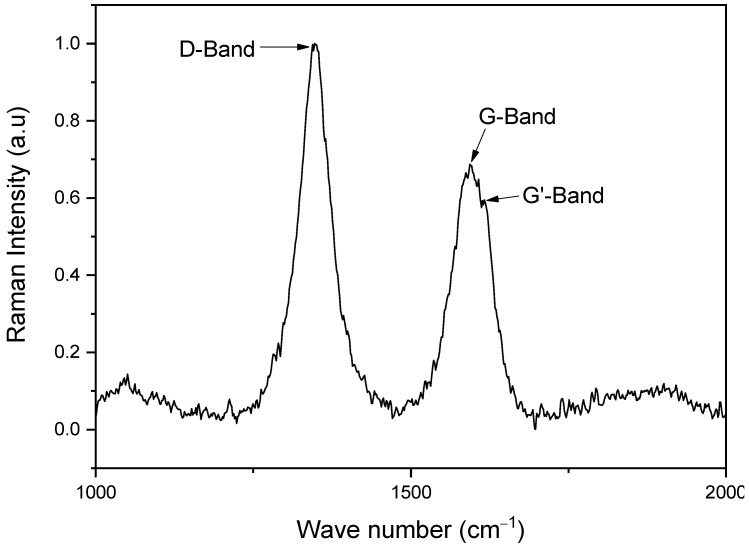
Raman spectrum of MWCNTs.

Figure 5 presents the Raman spectrum of the MWCNT dispersion. This spectrum reveals the presence of the D and G bands, as well as a G’ band that, although weak, has an intensity comparable to other G’ bands of sp^2^-bonded carbons cited in the literature [17]. The D band (at approximately 1348 cm^−1^) is associated with defects in the carbon structure, where additional vibrational modes are generated due to electron delocalization. The presence of this band indicates that there are perturbations in the carbon network, such as the formation of vacancies or the presence of impurities, whereas the presence of the G band (at approximately 1593 cm^−1^) is representative of an ordered carbon and is related to the vibration of sp^2^ bonds. A further increase in the intensity of this band suggests a greater order in the structure of the nanotubes. To estimate the structural order, the I_D_/I_G_ ratio is taken into account; to calculate this ratio, the Lorentzian probability function process is applied for the D and G bands, thus obtaining the position of the peaks [15].

The Raman spectra of the MWCNTs were analyzed using the ID/IG ratio, and a value of 1.451 was obtained, which is greater than 1, indicating that the intensity of the D band is greater than that of the G band, which implies a moderate number of defects in the structure of the nanotubes and, consequently, a degree of disorder in the dispersion [26]. These findings are consistent with previous research [15], which indicates that, at this surfactant concentration, an adequate balance is achieved between the dispersion of the nanotubes and the disorder induced in the system, as well as the defects generated by the applied dispersive energies.

Furthermore, this result is consistent with the proposed application, since the presence of defects can increase the surface reactivity of the nanotubes, favoring their interaction with the corrosive medium and improving their effectiveness as inhibitors. These defects can facilitate the adsorption of inhibitory molecules on the surface of the nanotubes, increasing their ability to prevent corrosion by forming an effective barrier between the metal and the corrosive medium.

### 3.2. UV-Vis Spectroscopy

**Figure 6:**
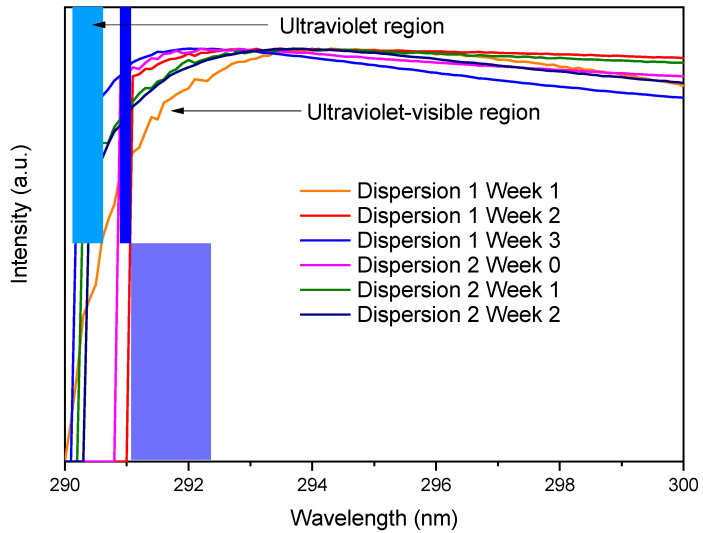
Evolution of UV-Vis absorption spectra of both dispersions during three weeks (own elaboration).

Figure 6 shows the UV-Vis absorption spectra of the dispersions of week 1 and 3, both for the reference dispersion (dispersion 1) and for the working dispersion (dispersion 2). This technique was used to evaluate the degree of dispersion of the carbon nanotubes (CNTs) in the solution. The agglomerated CNTs show absorption peaks in the ultraviolet region, while the individual CNTs show absorption peaks in the ultraviolet–visible region [27]. The absorption intensity is directly related to the degree of dispersion; the presence of absorption peaks in the UV-Vis region indicates that there is no significant amount of CNT agglomerations in the suspension.

In this graph, an absorption peak around 291 nm is observed, suggesting a good dispersion of the MWCNTs after one week of storage. Likewise, the absorption spectra of both dispersions are presented for three weeks, where the peak remains at approximately 291 nm wavelength. This shows that the dispersion remains stable for up to two weeks after its preparation. In this way, it is ensured that the dispersions remained stable at the time of preparing the cement specimens.

The maximum absorption value is observed around 291 nm for both dispersions during the three weeks of study, before they lose their stability. This absorption maximum is explained by the interaction between the MWCNTs and TX-100, which involves two main mechanisms:

1. π-π stacking: The benzene ring presented in TX-100 allows π-π stacking interactions with the surface of the MWCNTs [28]. This produces a reinforcement in the bond between the surfaces of the MWCNTs and TX-100, forming a stable shell [15].

2. Van der Waals forces: These forces are generated due to the molecular interactions between the alkyl chains of the surfactant and the carbon surface of the nanotubes. As a result, an assembled layer of TX-100 molecules is formed on the surface of the MWCNTs [29].

The combination of these two mechanisms contributes to the stability of the dispersion and explains the behavior observed in the UV-Vis absorption spectrum.

### 3.3. Zeta Potential

**Figure 7:**
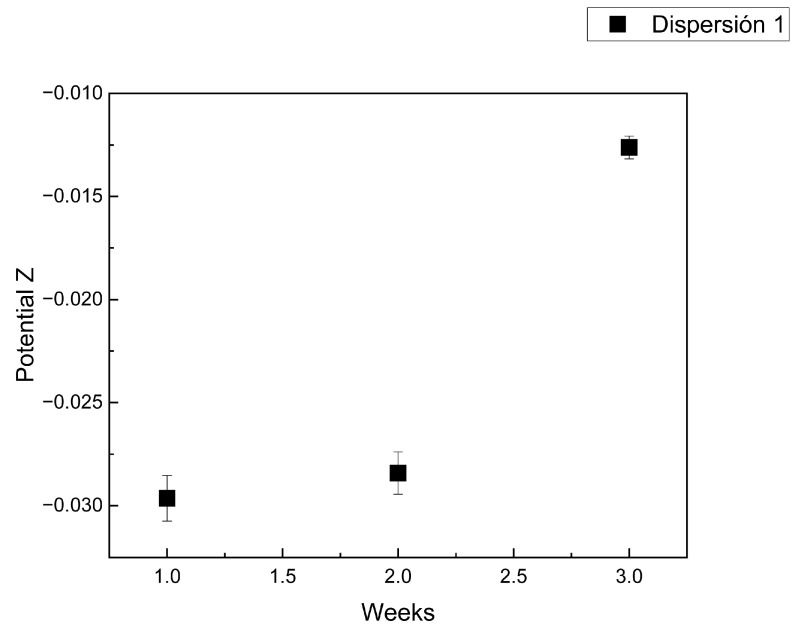
The Z potential of the first dispersion (own elaboration).

Figure 7 shows the trend of the Zeta potential of the reference dispersion (dispersion 1) over three weeks. During this time, a drop in potential was detected, reflecting the loss of stability in the dispersion due to the agglomeration of the nanotubes [15].

Initially, a good dispersion was observed, since the values were outside the range of instability reported in the literature (−25 mV to +25 mV) for carbon nanotube dispersions. The dispersion remained stable for a considerable time thanks to the effect of the surfactant at a concentration determined in previous studies [15]. It should be noted that, thanks to the non-ionic nature of the surfactant, it does not interfere with the repulsive charges of the carbon nanotubes and, therefore, does not affect the measurement of the Zeta potential.

The applied energy destabilizes the system and generates the dispersion, reflecting a very negative potential, indicating a strong electrostatic repulsion between the particles. When the potential approaches zero, the repulsion weakens, facilitating particle agglomeration and compromising the stability of the solution [30].

The reference dispersion (dispersion 1) was time-based monitored to determine the optimal moment of preparation of the specimens. When this dispersion falls into the region of unstable potential for MWCNTs, the working dispersion (dispersion 2) is used. A measurement of the Z potential of the latter is carried out at the time of its preparation to ensure a good dispersion before using it in the preparation of the cement specimens. A value of −42 mV was obtained, a potential considerably far from the range that indicates instability in the dispersion, thus guaranteeing optimal conditions for the preparation of the samples.

### 3.4. Linear Polarization Resistance (LPR)

**Figure 8:**
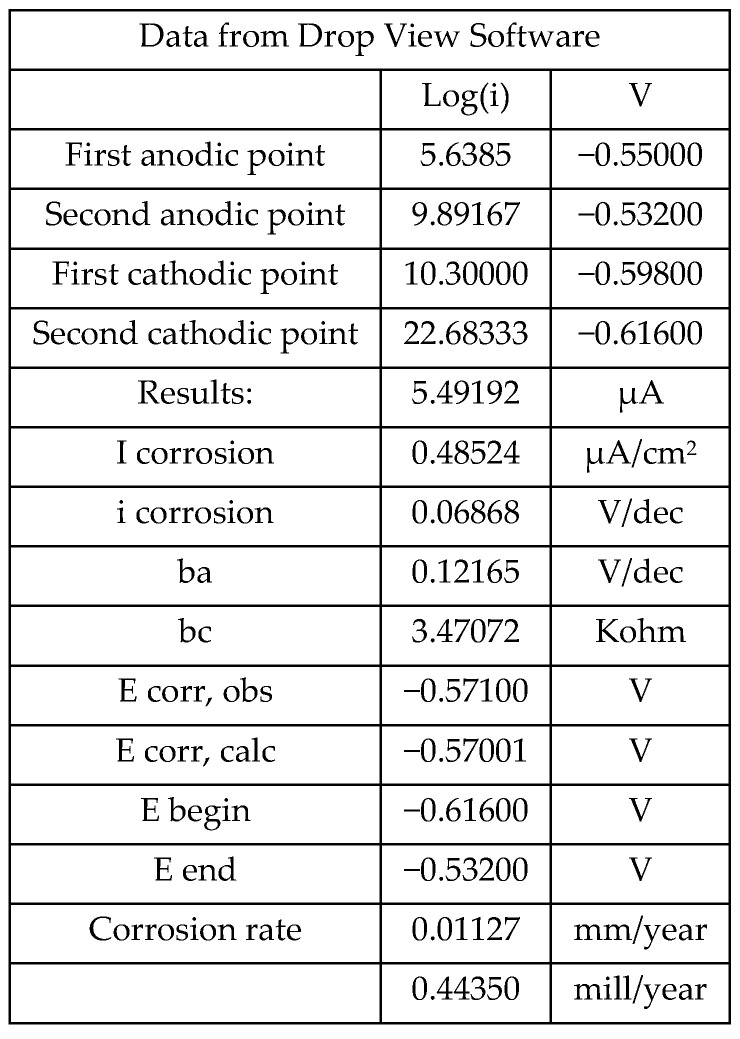
Data from Drop View software (TX-100 14 days second measurement, Drop View 8400).

**Figure 9:**
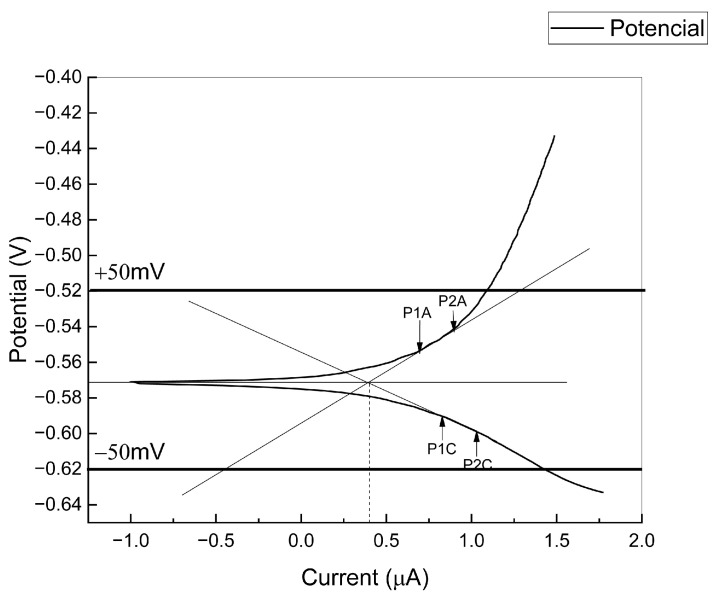
Tafel extrapolation (TX-100 14 days second measurement).

The calculation of the corrosion rate was performed using the potentiostat Drop View software, by setting the surface area, equivalent weight and density parameters (Figure 8). Figure 9 shows the Tafel curves obtained from the linear polarization resistance (LPR) measurements of the specimens.

The analysis process was carried out as follows:

1. The Tafel extrapolation curve was generated by plotting the logarithm base 10 of the absolute value of the recorded current versus the potential.

2. Four points were selected on this curve, within a range of ±50 mV around the corrosion potential. This interval was chosen because, in this region, the cathodic and anodic processes contribute almost entirely to the measured current [31].

3. These points were used to determine the slopes of the potential in the anodic (rising) and cathodic (falling) regions.

4. Based on these data, the software calculated the corrosion rate and other relevant parameters.

**Figure 10:**
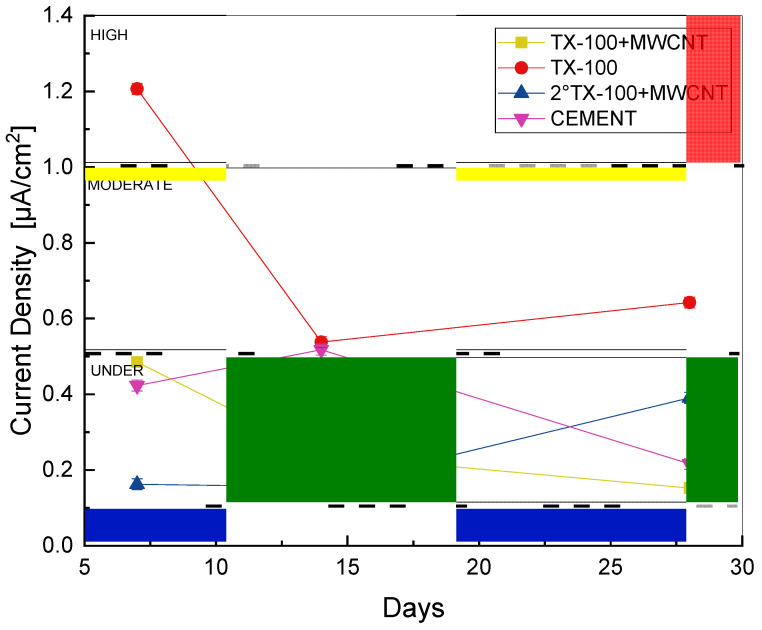
Current density vs. time of reinforcing steel in all cement specimens.

Figure 10 shows the corrosion rate values, expressed in μA/cm^2^, of the different cement specimens that varied in composition, i.e., TX-100 + MWCNTs: test pieces made with the first dispersion; TX-100: test pieces made with only the surfactant; TX-100 + MWCNTs: test pieces made with the second dispersion; and CEMENT: test pieces without any additives.

These data were obtained using the linear polarization resistance (LPR) technique, considering the sensitivity of the equipment according to its technical data sheet (0.025% of the range of the measured current) for the standard deviation of the measured data.

Analyzing Figure 10 and considering Table 1, which expresses the corrosion risk as a function of the corrosion current density in µA/cm^2^, the specimen characteristics that can be identified are as follows: (i) The specimens made with the dispersions show a lower corrosion risk than those made with the surfactant. (ii) The specimens with only surfactant present the highest corrosion risk, between moderate and very high. This is associated with the pore size, since, due to the characteristics of the surfactant, the air is trapped in the cement matrix, generating larger pores and crack propagation, a consequence of its soapy texture. Finally, (iii) the specimens made with MWCNT dispersions, compared to the cement specimens without additive, show a behavior in terms of corrosion risk, being more stable throughout the study.

The results obtained for cement without additives, which indicate a moderate-to-high risk of corrosion, are consistent with those reported in the literature. A specific study evaluated this property in cement specimens prepared using three curing methods: immersion, membrane and steam. Similar corrosion levels were observed in two of these methods, including immersion curing. These results coincide with those obtained in the present study [32].

**Table 1:** Corrosion risk according to corrosion rate (taken from [33]).

i_corr_ µA/cm^2^	V_corr_ mm/year	Corrosion Level
≤0.1	≤0.001	Despicable
0.1–0.5	0.001–0.005	Low
0.5–1	0.005–0.01	Moderate
>1	>0.01	High

The analysis of the corrosion risk over the curing time revealed significant trends in the specimens studied. After seven days of curing, the second dispersion showed a lower risk of corrosion compared to the other specimens. After fourteen days, both dispersions exhibited a more favorable behavior, presenting a lower risk of corrosion than the specimens without additives. After twenty-eight days of curing, both dispersions were in a low corrosion risk range. This favorable behavior can be attributed to the ability of carbon nanotubes to reduce porosity in the cement matrix, thanks to the fineness of their particles and their homogeneous distribution [13], as evidenced in the SEM images.

This reduction in porosity could prevent corrosive agents from altering the passivity of the protective layer of the reinforcing steel, thus preventing the chemical reaction that triggers corrosion. At the end of this experimental study, it was observed that both dispersions maintained a low corrosion risk, suggesting that the addition of carbon nanotubes does not compromise the natural resistance of cement to corrosion. This intrinsic resistance is due to the capacity of cement to consume free chlorides by combining them chemically or physically, as it reacts with the products of hydration of the ceramic material such as tricalcium aluminate 3CaO*Al_2_O_3_ (C3A) that forms chlorine aluminates. Also, when tetracalcium ferroaluminate (CAFA) reacts, it forms calcium chlorine ferrite. Similarly, the reaction of chlorides when corroding steel contributes to their decrease in the electrolyte [34].

It is crucial to consider that the presence of free chloride ions directly influences the corrosion current density, since these ions are essential for the corrosion process in steel embedded in this corrosive medium.

### 3.5. SEM

**Figure 11:**
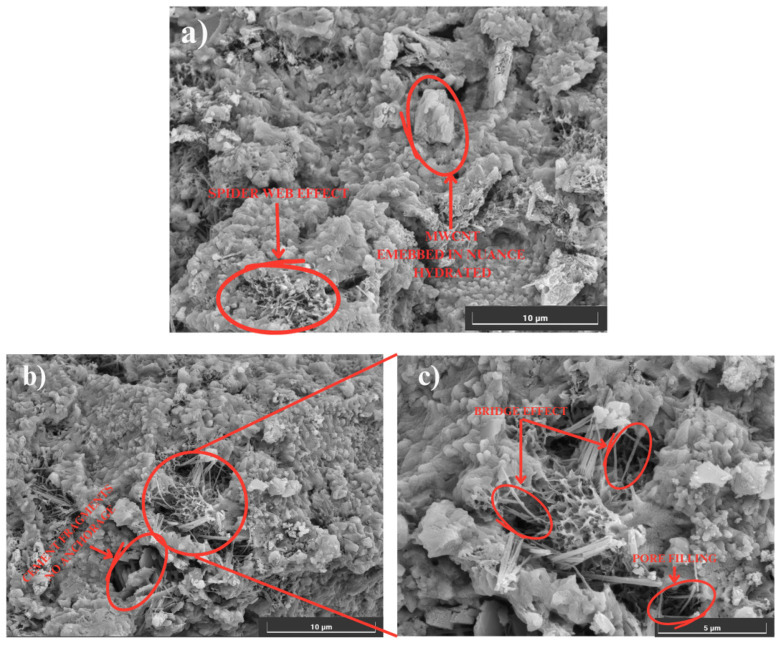
SEM. (**a**) Cement + MWCNT at 10 μm. (**b**) Cement + MWCNT at 10 μm. (**c**) Cement + MWCNT at 5 μm.

In Figure 11a–c, we observe the microscopy of the specimens with MWCNTs at a scale of 10 μm and 5 μm, where different forms of arrangement of MWCNTs embedded in a hydrated matrix are evident: the bridge effect, where a MWCNT is anchored by its ends to two fragments of cement; the spider web effect, which is a grouping zone of the MWCNTs; and, finally, the arrangement of the MWCNTs grouped in the pores of the cement matrix, generating a “filling” in them [15].

**Figure 12:**
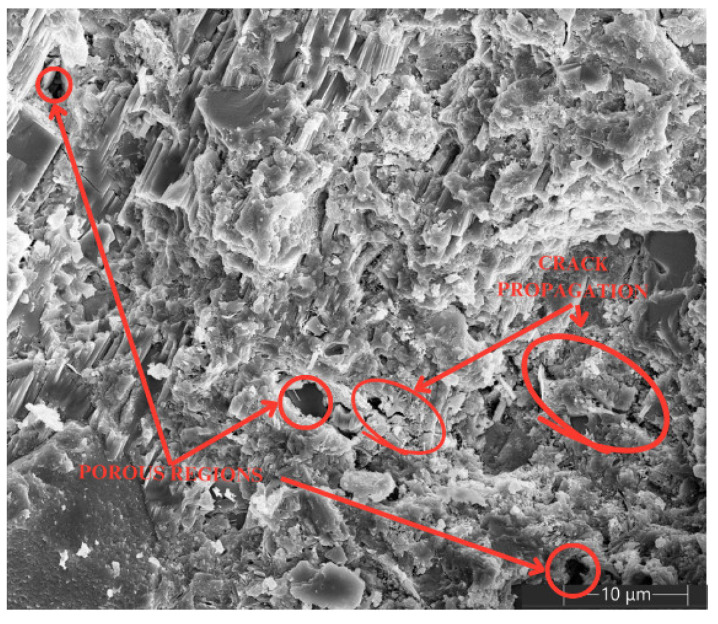
SEM. Cement at 10 μm.

**Figure 13:**
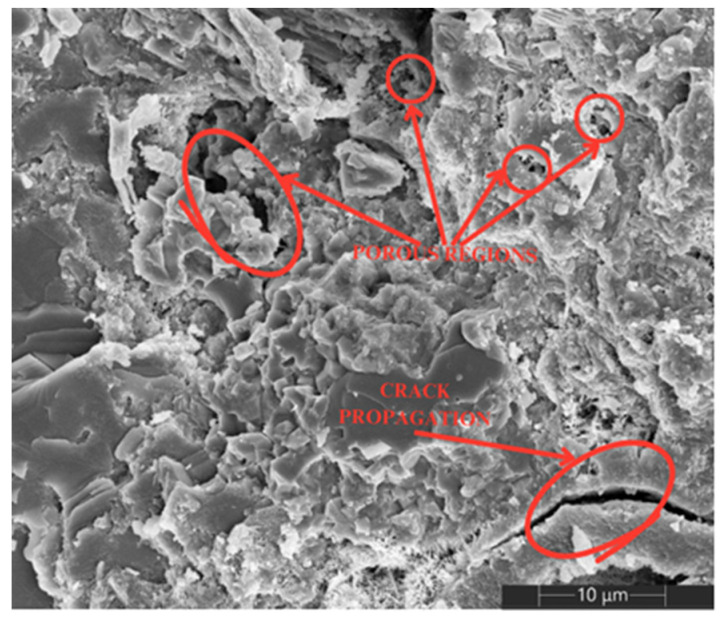
SEM. Cement + TX-100 at 10 μm.

Additionally, Figure 12 and Figure 13 show the microscopy of the specimens without the addition of MWCNTs, where Figure 12 corresponds to the specimen composed only of cement and Figure 13 corresponds to a specimen of cement with TX-100. In both images, completely empty pores and crack propagation can be observed; however, in the cement and TX-100 specimen, the propagation of cracks and the formation of completely empty pores of different sizes, visible in the SEM images, associated with the retention of gasses in the mixture during the specimen preparation process, caused by the surfactant nature of TX-100, are more visible.

Correlating the results shown on the SEM images with those obtained in the calculation of the corrosion rate can explain why the current density is higher in the specimens with TX-100. Due to the presence of different pore sizes and longer cracks, the cement and TX-100 specimens provide spaces through which chloride ions infiltrate directly until they reach the embedded steel, which generates the loss of the passive oxide layer that protects the metal and starts the corrosion process. On the other hand, in the specimens with MWCNTs, in addition to having a smaller number of defects, the incorporation and grouping of MWCNTs in the aforementioned dispositions can be seen. According to the literature, due to Van der Waals forces, MWCNTs interfere with the intrusion of chloride ions in the cementitious matrix, which favors the conservation of the passive layer of the embedded steel and maintains a low corrosion risk.

## 4. Conclusions

This study established the influence of multi-walled carbon nanotubes (MWCNTs) on the corrosion resistance of Portland cement embedded steel, unveiling the following main findings:

Dispersion stability: MWCNT dispersions prepared with TX-100 at a concentration of 10 mM and a sonication energy of 440 J/g remained stable for three weeks. This ensured a homogeneous distribution of the nanotubes in the cement matrix, a key factor in improving the strength of the material.

Electrochemical behavior: Linear polarization resistance tests demonstrated that the addition of MWCNTs significantly reduced the corrosion rate of the embedded steel, acting as a barrier against chloride ion intrusion. This effect was more evident in dispersions with a shorter storage time, highlighting the importance of stability in the preparation of dispersions.

Structural characterization: Raman spectroscopy revealed an equilibrium between the dispersion of MWCNTs and the presence of structural defects in the nanotubes, which favored their interaction with corrosive agents, improving their capacity as corrosion inhibitors.

Internal structure of the cement: Scanning electron microscopy (SEM) allowed the observation of three distinctive behaviors in the cement matrix: agglomeration of MWCNTs around hydrated pores, formation of structural bridges and formation of homogeneous dispersion around the pores. These characteristics contributed to the reduction in porosity and the improvement in the mechanical properties of the cement.

These findings not only confirm the ability of MWCNTs to strengthen cement, but also highlight their potential to increase the protection of embedded steel against corrosion. These results open new perspectives for research in nanomaterials as reinforcement in construction applications, with the potential to extend the lifespan of structures and reduce long-term maintenance costs.

## Data Availability

The original contributions presented in this study are included in the article. Further inquiries can be directed to the corresponding author(s).

## References

[1] Gómez E., Aldrhy M. (2019). Evaluación Electroquímica de la Interacción Acero-Concreto Debido a la Introducción de Nanopartículas Base Silicio a Través de un Tratamiento Superficial. Master’s Thesis.

[2] Luján F., Alberto V. (2020). Efecto de la Corrosión por Ión Cloruro en las Propiedades Mecánicas de Acero de Refuerzo Embebido en Concretos Ternarios CP-CV-CBC.

[3] (2020). Técnicas Aceleradas para Evaluar la Susceptibilidad a Corrosión de Aceros Embebidos en Morteros con Adiciones Minerales Expuestos a Cloruros. Informador Técnico. https://revistas.sena.edu.co/index.php/inf_tec/article/view/158.

[4] Song H.W., Jung M.S., Lee C.H., Kim S.H., Ann K.Y. (2010). Influence of Chemistry of Chloride Ions in Cement Matrix on Corrosion of Steel. ACI Mater. J..

[5] Cárdenas M.A. La Carbonatación del Concreto… Un Problema en un Mundo Industrializado. LinkedIn. https://es.linkedin.com/pulse/la-carbonataci%C3%B3n-del-concreto-un-problema-en-mundo-alvarez-cardenas.

[6] Xu J., Jiang L., Wang J. (2009). Influence of Detection Methods on Chloride Threshold Value for the Corrosion of Steel Reinforcement. Construction and Building Materials.

[7] Sagoe-Crentsil K.K., Glasser F.P. (1993). “Green rust”, iron solubility and the role of chloride in the corrosion of steel at high pH. Cem. Concr. Res..

[8] Konsta-Gdoutos M.S., Metaxa Z.S., Shah S.P. (2010). Highly dispersed carbon nanotube reinforced cement-based materials. Cem. Concr. Res..

[9] Giraldo J. (2015). Efecto de la Introducción de Nanopartículas Sobre la Microestructura de Matrices Endurecidas Base Cemento Portland. Master’s Thesis.

[10] Torres R., Aperador W., Vera E., Mejía R., Ortiz C. (2010). Estudio de la Corrosión del Acero Embebido en Concreto AAS Sometido a Cloruros.

[11] Holland R.B., Kurtis K.E., Kahn L.F. (2016). Effect of different concrete materials on the corrosion of the embedded reinforcing steel. En Corrosion of Steel in Concrete Structures.

[12] Flores A., Uruchurtu J. (2021). Efecto de la Corrosión del Concreto Reforzado con Adición de Polvo de Grafito y su Evaluación en sus Propiedades Físicoelectroquímicas.

[13] Marcondes C.G.N., Medeiros M.H.F. (2016). Análisis de la dispersión de soluciones conteniendo nanotubos de carbono para su uso en concretos de cemento Portland. Rev. Alconpat..

[14] Hielscher. Nano Particles. Hielscher Ultrasonics. https://www.hielscher.com/es/nano_03.htm.

[15] Echeverry L. (2020). Energías de Dispersión y Surfactantes en una Solución de Nanotubos de Carbono en Agua: Aplicaciones en Pastas de Cemento Portland. Master’s Thesis.

[16] Parveen S., Rana S., Fangueiro R. (2013). A Review on Nanomaterial Dispersion, Microstructure, and Mechanical Properties of Carbon Nanotube and Nanofiber Reinforced Cementitious Composites. J. Nanomater..

[17] García González M.A., Sánchez Mendonza E., Waksman Minsky N., Saucedo A.L. (2022). Fundamentos y analogías para entender mejor la espectroscopía de RMN. Educ. Química.

[18] Concepción D., Gonzalo S. (2007). Title of Chapter. Espectroscopía Raman de Nanotubos de Carbono.

[19] Alberto R.R.M. Caracterización Estructural y Óptica de Nanotubos de Carbón Funcionalizados con NI y AG. http://zaloamati.azc.uam.mx/handle/11191/5750.

[20] Hanako R.S. Microscopía Electrónica de Barrido y Microanálisis de Elementos del Clúster Científico y Tecnológico BioMimic. INECOL. https://www.mexicoambiental.com/microscopia-electronica-de-barrido-y-microanalisis-de-elementos-del-cluster-cientifico-y-tecnologico-biomimic/.

[21] Pérez J.B. Diseño y Construcción de un Prototipo de Potenciostato Galvanostato para el Laboratorio de Corrosión de la Escuela de Ingeniería Metalúrgica. Dialnet. https://dialnet.unirioja.es/servlet/articulo?codigo=6299760.

[22] Terán J., Arroyo M., Teresa M.V., Vázquez D. (2022). Desarrollo de un Sistema Galvanostático para la Corrosión Acelerada en Varillas de Refuerzo.

[23] Chousidis N., Zacharopoulou A., Zeris C., Batis G. (2022). Corrosion resistance and physical-mechanical properties of reinforced mortars with and without carbon nanotubes. J. Mater. Sci. Chem. Eng..

[24] Icontec (2021). Norma Técnica Colombiana NTC 1377: Ingeniería Civil y Arquitectura. Elaboración y Curado de Especímenes de Concreto para Ensayos de Laboratorio.

[25] Icontec (2020). Norma Técnica Colombiana NTC 550: Concretos. Elaboración y Curado de Especímenes de Concreto en el Sitio de Trabajo.

[26] Ferrari A.C., Robertson J. (2000). Interpretation of Raman spectra of disordered and amorphous carbon. Phys. Rev. B Condens. Matter.

[27] Ryabenko A.G., Dorofeeva T.V., Zvereva G.I. (2004). UV–VIS–NIR spectroscopy study of sensitivity of single-wall carbon nanotubes to chemical processing and Van-der-Waals SWNT/SWNT interaction. Carbon.

[28] Li H., Qiu Y. (2019). Dispersion, sedimentation and aggregation of multi-walled carbon nanotubes as affected by single and binary mixed surfactants. R. Soc. Open Sci..

[29] Di Crescenzo A., Di Profio P., Siani G., Zappacosta R., Fontana A. (2016). Optimizing the interactions of surfactants with graphitic surfaces and clathrate hydrates. Langmuir ACS J. Surf. Colloids.

[30] Alsharef J.M.A., Taha M.R., Al-Mansob R.A., Govindasamy P. (2019). Evaluation of the Dispersion Stability of Nanocarbons Using Zeta Potential in Distilled Water. Nano Hybrids Compos..

[31] Rocchini G. (1994). The determination of tafel slopes by an integral method. Corros. Sci..

[32] Estrada A., López A., Chavarría M., Rojas Z. (2014). Velocidad de Corrosión en el Sistema Acero-Concreto: Enfoque al Método de Curado.

[33] Díaz A., Arganis C., Torres A., Rodriguez A., Valle A., Robles E., Pérez J., Rincón O. (2019). Determinación de la Velocidad de Corrosión de Varillas en Probetas de Concreto Armado.

[34] Pedraza P.A. (2007). Estudio Preliminar del Comportamiento Frente a la Corrosión de Varillas de Acero Embebido en Concreto de Escoria Activada Alcalinamente. Master’s Thesis.

